# Pleiotrophin regulates microglia-mediated neuroinflammation

**DOI:** 10.1186/s12974-017-0823-8

**Published:** 2017-03-04

**Authors:** Rosalía Fernández-Calle, Marta Vicente-Rodríguez, Esther Gramage, Jimena Pita, Carmen Pérez-García, Marcel Ferrer-Alcón, María Uribarri, María P. Ramos, Gonzalo Herradón

**Affiliations:** 1 Pharmacology Lab, Department of Pharmaceutical and Health Sciences, Facultad de Farmacia, Universidad CEU San Pablo, Urb. Montepríncipe, 28668 Boadilla del Monte, Madrid Spain; 2 Biochemistry and Molecular Biology lab, Department of Chemistry and Biochemistry, Facultad de Farmacia, Universidad CEU San Pablo, Madrid, Spain; 3 BRAINco Biopharma, S.L., Bizkaia Technology Park, Vizcaya, Spain

**Keywords:** Microgliosis, Microglia activation, Midkine, Neuroimmune response, Neuroinflammation, Pleiotrophin, TLR4

## Abstract

**Background:**

Pleiotrophin (PTN) is a cytokine found highly upregulated in the brain in different disorders characterized by overt neuroinflammation such as neurodegenerative diseases, drug addiction, traumatic injury, and ischemia. In the present work, we have explored whether PTN modulates neuroinflammation and if Toll-like receptor 4 (TLR4), crucial in the initiation of an immune response, is involved.

**Methods:**

In immunohistochemistry assays, we studied lipopolysaccharide (LPS, 7.5 mg/kg i.p.)-induced changes in glial fibrillary acidic protein (GFAP, astrocyte marker) and ionized calcium-binding adaptor molecule 1 (Iba1, microglia marker) expression in the prefrontal cortex (PFC) and striatum of mice with transgenic PTN overexpression in the brain (PTN-Tg) and in wild-type (WT) mice. Cytokine protein levels were assessed in the PFC by X-MAP technology. The influence of TLR4 signaling in LPS effects in both genotypes was assessed by pretreatment with the TLR4 antagonist (TAK-242, 3.0 mg/kg i.p.). Murine BV2 microglial cells were treated with PTN (0.5 μg/ml) and LPS (1.0 μg/ml) and assessed for the release of nitric oxide (NO).

**Results:**

We found that LPS-induced microglial activation is significantly increased in the PFC of PTN-Tg mice compared to that of WT mice. The levels of TNF-α, IL-6, and MCP-1 in response to LPS were significantly increased in the PFC of PTN-Tg mice compared to that of WT mice. Pretreatment with TAK-242 efficiently blocked increases in cytokine contents in a similar manner in both genotypes. Concomitant incubation of BV2 cells with LPS and PTN significantly potentiated the production of NO compared to cells only treated with LPS.

**Conclusions:**

Our findings identify for the first time that PTN is a novel and potent regulator of neuroinflammation. Pleiotrophin potentiates LPS-stimulated microglia activation. Our results suggest that regulation of the PTN signaling pathways may constitute new therapeutic opportunities particularly in those neurological disorders characterized by increased PTN cerebral levels and neuroinflammation.

## Background

Inflammation is a key event in the healing process of the damaged tissue, and activation of the innate immune system is fundamental in the response to inflammation. When prolonged, however, inflammation can become deleterious. Within the central nervous system (CNS), the two main players in neuroinflammation are glial cells: microglia, the resident macrophages in the CNS, and astrocytes [1]. Toll-like receptors (TLRs), which are expressed in the rodent microglia and astrocytes [2], are key molecules in the activation of innate immunity during CNS damage. Activation of TLRs triggers the downstream stimulation of nuclear factor-κB (NFκB) and the induction of genes that encode inflammation-associated molecules and cytokines including TNF-α, IL-1β, IL-6, iNOS, and COX2 [2–4].

Ever-growing evidence points to a key role of inflammatory processes in a broad spectrum of diseases including traumatic brain injury, chronic neurodegenerative diseases, neuropathic pain, ischemia, and neuropsychiatric disorders including drug addiction [5–7]. Psychostimulants such as amphetamine and its derivatives cause neuroinflammation and limit neurogenesis and induce blood-brain barrier (BBB) damage [8, 9]. All these effects induced by amphetamines are important for the dopaminergic injury induced by these drugs in the nigrostriatal pathway [10], which is the same circuitry affected in Parkinson’s disease (PD). Interestingly, recent evidence indicate a 3-fold increased risk of PD in these drug addicts [11], suggesting that common pathogenic mechanisms could underlie both diseases, PD and amphetamines abuse, one of which could be neuroinflammation [9, 12]. In the search for validation of new biomarkers and for the development of new drugs that could modulate the inflammatory processes underlying these and other diseases of the CNS [13], our strategy was to identify proteins with known regulatory functions in inflammation, whose levels of expression are upregulated after amphetamine administrations and in the neurodegenerative areas of the brain of PD patients.

Pleiotrophin (PTN) is a cytokine that is upregulated in different brain areas after administration of different drugs of abuse including amphetamine [14, 15] and in the nigrostriatal pathway of patients with PD [16]. PTN is important for CNS repair and for survival and differentiation of dopaminergic neurons [6]. In addition, evidence points to a modulatory role of PTN on inflammation. In peripheral organs, PTN is known to induce inflammatory mediators [17] and its expression levels are significantly reduced by administration of anti-inflammatory drugs [18]. Little is known about a possible role of PTN on neuroinflammation. Transgenic mice with PTN overexpression in the brain (PTN-Tg) show a ~4-fold increased amphetamine-induced striatal astrogliosis compared to wild-type (WT) mice [19]. However, PTN genetically deficient (PTN-/-) mice show a modest ~20% increase in amphetamine-induced astrocytosis, possibly reflecting proinflammatory compensatory mechanisms [19–21]. We hypothesize that PTN is a novel modulator of neuroinflammation. To test this hypothesis, we have now comparatively studied the astrocytic response, microglial activation, and cytokine release induced by lipopolysaccharide (LPS) in PTN-Tg and in WT mice. Since previous studies suggest a possible contribution of PTN to Toll-like receptor 4 (TLR4)-mediated immune response [22] and TLR4 plays a pivotal role in neuroinflammation, we have also investigated the possible differential contribution of TLR4 to the neuroinflammatory processes induced by LPS in both genotypes.

## Methods

### Animals

PTN-Tg mice on a C57BL/6J background were generated by pronuclear injection as previously described [23, 24]. The acceptor vector contained the regulatory regions responsible for tissue-specific expression of Thy-1 gene, which drives neuron-specific expression of transgenes [25, 26]. PTN-specific overexpression in different brain areas, including an ~3–4-fold upregulation in the prefrontal cortex (PFC) and a 20% increase of PTN protein levels in striatum, was established by quantitative real-time polymerase chain reaction (qRT-PCR), in situ hybridization, and by western blot [23, 27, 28].

We used male PTN-Tg and WT animals of 9–10 weeks (20-25 g). Mice were housed under controlled environmental conditions (22 ± 1 °C and a 12-h light/12-h dark cycle) with free access to food and water.

All the animals used in this study were maintained in accordance with the European Union Laboratory Animal Care Rules (86/609/ECC directive), and the protocols were approved by the Animal Research Committee of USP-CEU.

### Treatments

To test genotypic differences in LPS-induced neuroinflammation and the involvement of TLR4 in LPS effects in PTN-Tg and WT mice, we assessed the effects of LPS in mice pretreated with the TLR4 antagonist TAK-242 (Merck Millipore, Madrid, Spain). For this purpose, mice were injected (i.p.) with TAK-242 (3 mg/kg) or saline (10 ml/kg; control) 30 minutes before a single i.p. injection of LPS (7.5 mg/kg) or saline (10 ml/kg; control). As a result, we obtained four experimental groups in both genotypes: saline + saline (Sal-Sal), saline + LPS (Sal-LPS), TAK-242 + saline (TAK-Sal), and TAK-242 + LPS (TAK-LPS). For immunohistochemistry analysis, animals were sacrificed 16 h after LPS or last saline administration (*n* = 4–5/group/genotype) by perfusion with 4% p-formaldehyde. For tisular analysis of cytokine levels, animals were decapitated 16 h after LPS or the last saline administration (*n* = 5/group/genotype) and the PFC was rapidly removed and frozen in dry ice and stored to −80 °C until the protein extraction procedure.

### Immunohistochemistry analysis

Mice were transcardially perfused with 4% p-formaldehyde; the brains were removed and conserved in p-formaldehyde for 24 h and then transferred to a 30% sucrose solution containing 0.02% sodium azide for storage at 4 °C; 30-μm PFC and striatal free-floating sections were processed as previously described [20, 21, 29]. Immunohistochemistry studies were performed in one slice per 180 μm (the PFC from the bregma −3.08 mm to −2.46 mm; the striatum from the bregma 1.54 mm to −0.10 mm).

In order to study gliosis, sections were incubated overnight at 4 °C with anti-glial fibrillary acidic protein (GFAP; Millipore, Madrid, Spain; 1:1000) and anti-ionized calcium-binding adaptor molecule 1 (Iba1, Wako, Osaka, Japan; 1:1000) antibodies, following by 30-min incubation with the Alexa-Fluor-555 and Alexa-Fluor-488 corresponding secondary antibody (Invitrogen, Waltham, MA, USA; 1:500). Sections were mounted on gelatin-coated slides and coverslipped with Fluoromount medium. Photomicrographs were captured with a digital camera coupled to an optical microscope (DM5500B, Leica, Solms, Germany). Analysis was performed using ImageJ (NIH, Bethesda, MD, Version 1.50f), in the three most central slices of each area. Iba1+ cells and GFAP+ astrocytes were counted in whole sections of the PFC and in 1100 μm × 1400 μm standardized areas in the striatum as previously described [21, 29]. In the case of Iba1-ir, total marked area was calculated as overall image fluorescence, subtracting the mean background fluorescence. Computer-based analysis of the morphology of individual Iba1+ cells was performed using the “Analyze Particle” function in ImageJ software. Images were segmented and smoothed to best fit cell shape. To be sure to select only cells entirely present in the acquired field, cells with an area of >25 μm^2^ were analyzed. The objects meeting the minimum size to be analyzed were measured for the following parameters: area, perimeter, and circularity. The soma size (cell area) is expressed in square micrometers. The perimeter was calculated based on the outline length of a given object. It measures the length around the periphery of each soma and is expected to be higher in activated and hypertrophic cells. Circularity was calculated by the following formula: 4π × (area/perimeter^2^). This parameter varies from 0 (linear polygon) to 1 (perfect circular object). Mean single-cell values for each parameter were used for statistics.

### Cytokine levels

Approximately 5 mg of each sample of the PFC (*n* = 5/group) was homogenized in 100 μl of homogenization buffer (0.05% Tween 20 and protease inhibitor cocktail (Thermo Fisher Scientific Inc., Waltham, MA, USA) in PBS, pH 7.2). After 3 cycles of 1 min and 50 Hz in the tissuelyzer (Quiagen, Germantown, Maryland, USA), samples were centrifuged at 11000×*g* for 30 min at 4 °C. After centrifugation, supernatants were transferred to a new tube and stored at −80 °C until the assay. Total protein content of each sample was measured using the BCA protein assay kit (Thermo Fisher Scientific Inc., Waltham, MA, USA). Levels of tumor necrosis factor-α (TNF-α), interleukin 1β (IL-1β), interleukin 6 (IL-6), monocyte chemoattractant protein-1 (MCP-1), interleukin 4 (IL-4), and interleukin 10 (IL-10) were measured by X-Map technology using a Milliplex MADPK-71K adipokine kit according to the manufacturer’s description (Merck Millipore, Spain).

### BV2 cell cultures

#### BV2 murine microglial cells

BV2 murine microglial cells were a generous gift from Professor Antonio Cuadrado (Instituto de Investigaciones Biomédicas “Alberto Sols” (IIBM), Madrid, Spain). Cells were routinely maintained in RPMI-1640 medium with fetal bovine serum (10%), penicillin (100 U/ml), streptomycin (100 μg/ml), and L-glutamine (4 mM) at 37 °C in 5% CO_2_ humidified air following conditions used by others [30, 31]. Prior to each experiment, cells were grown for 24 h on 96-well plates at a concentration of 1 × 10^4^ cells per well.

#### Measurement of NO production

Nitric oxide (NO) production was quantified by nitrite accumulation in the culture medium using the Griess reactive (2.25% sulfanilamide and 0.22% N-(1-naphthyl)-ethylenediamine dihydrochloride), according to protocols previously described [32, 33]. After fasting the cells for 24 h, BV2 cells were stimulated with different concentrations of PTN (0.05 μg/ml or 0.5 μg/ml), with or without LPS (1.0 μg/ml), for another 24 h. The concentrations of PTN were selected to correlate with the PTN overexpression in the brain of PTN-Tg mice and are within the range of concentrations used before to test the effects of PTN in neuronal injury [34] and in the expansion of human stem cells [35]. The NO production by cells was quantified in a microplate reader (Versa-Max, Molecular Devices, Sunnyvale, CA, USA) at 540 nm and then calculated with reference to the standard curve generated with NaNO_2_.

### Statistics

Data are presented as mean ± standard error of the mean (SEM). Data obtained from image analysis of the striatal and PFC immunostaining and cytokine levels were analyzed using two-way ANOVA considering genotype and treatment as variants. Relevant differences were analyzed by post hoc comparisons with Bonferroni’s post hoc tests. Data obtained from BV2 cells were analyzed using one-way ANOVA followed by post hoc comparisons with Tukey’s post hoc tests. *P* < 0.05 was considered as statistically significant. All statistical analyses were performed using Graph-Pad Prism program (San Diego, CA, USA).

## Results

### Differential regulation of LPS-induced astrocytic and microglial responses in the PFC of PTN-Tg mice. Effect of TAK-242 on LPS-induced microglial response and changes in cytokine contents in the PFC of PTN-Tg and WT mice

**Fig. 1:**
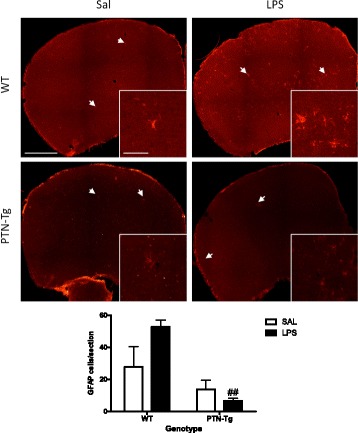
LPS effects on astrocytosis in the PFC of WT and PTN-Tg mice. Photomicrographs are from GFAP-immunostained PFC sections of saline + saline (Sal)- or saline + LPS (LPS)-treated animals (*n* = 4–5/group). Higher magnification images in the *lower right corner* of every representative picture show that astrocytes were hypertrophic and densely stained in WT mice treated with LPS. The graph represents quantification of data obtained from the counts of GFAP-positive cells in PFC whole sections. ^##^ *P* < 0.01 vs. WT-LPS. Scale bar = 500 μm, (high magnification, scale bar = 50 μm). *SAL* saline, *LPS* lipopolysaccharide, *WT* wild-type mice, *PTN-Tg* mice with transgenic pleiotrophin overexpression in the brain, *GFAP* glial fibrillary acidic protein

PTN is a cytokine highly upregulated in the brain in different CNS disorders characterized by neuroinflammation [6]. We aimed to study the modulatory role of PTN overexpression (3-fold) in the PFC of PTN-Tg mice [23, 24] on LPS-induced neuroinflammation. To investigate proinflammatory responses in these experiments, we tested the astrocytic and microglial response in PFC sections of WT and PTN-Tg mice treated with saline (Sal-Sal) or LPS (Sal-LPS). Astrocyte activation was assessed by morphology and expression of GFAP, an astrocyte-specific intermediate filament protein [36]. LPS treatment tended to increase the number of GFAP+ astrocytes in the PFC of WT mice, being these cells characterized by large densely stained cell bodies as well as long and extensive processes compared to saline-treated animals (Fig. 1). In contrast, these effects on GFAP+ IR induced by LPS were blocked in PTN-Tg mice (Fig. 1).

**Fig. 2:**
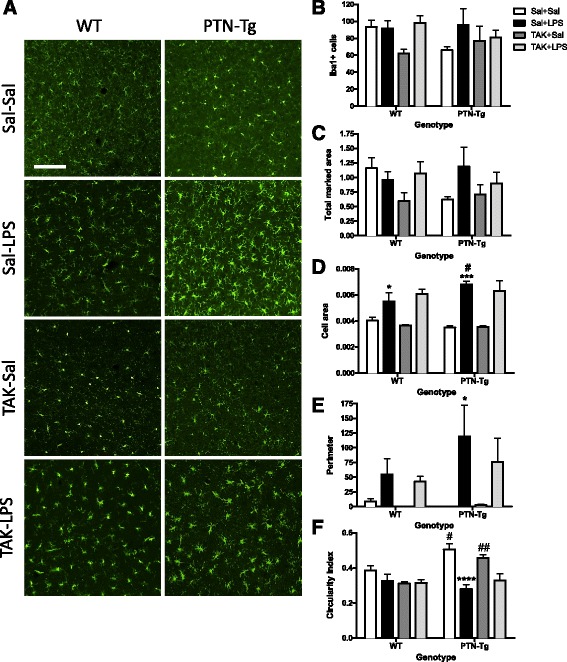
Effects of TAK-242 and LPS on microglia activation in the PFC of PTN-Tg mice. Photomicrographs are from Iba-1-immunostained PFC sections of saline (Sal)- or TAK-242 (TAK)-pretreated and saline (Sal)- or LPS-treated animals (*n* = 4–5/group) (**a**). Graphs represent quantification of data (mean ± SEM) obtained from the counts of Iba-1-positive cells (**b**), total marked area (**c**), cell area (**d**), soma perimeter (**e**), and circularity index (**f**) in PFC whole sections. *P < 0.05, ***P < 0.001, ****P < 0.0001 vs. Sal + Sal within the same genotype. ^#^ *P* < 0.05, ^##^ *P* < 0.01 vs. WT within the same treatment. Scale bar = 100 μm. *SAL* saline, *LPS* lipopolysaccharide, *TAK* TAK-242, *WT* wild-type mice, *PTN-Tg* mice with transgenic pleiotrophin overexpression in the brain, *Iba1* ionized calcium-binding adaptor molecule 1

Previous studies have linked LPS-induced activation of microglia and production of proinflammatory factors to brain damage and neurodegeneration [37]. To investigate proinflammatory responses in this experiment, PFC sections were immunostained with anti-Iba1 microglial antibody. In the saline control groups, microglial cells have resting morphology (Fig. 2a, Sal-Sal). Immunohistochemistry for Iba1 did not reflect significant changes in the number of Iba1+ cells in the PFC of WT and PTN-Tg mice after LPS treatment (Fig. 2b). However, we observed a clearly enhanced hypertrophism characterized by activation, soma enlargement, and sprouting of new ramifications in LPS-treated PTN-Tg mice compared to that in LPS-treated WT mice and saline-treated mice from the same genotype (Fig. 2a, Sal-LPS). Accordingly, the total marked area (Fig. 2c) tended to increase in LPS-treated PTN-Tg mice compared to that in saline-treated mice. As expected, the Iba1+ cell area was increased in LPS-treated mice of both genotypes compared with that in saline-treated mice (Fig. 2d); however, LPS induced an increase in cell area that was significantly enhanced in PTN-Tg mice compared to that in WT mice (Fig. 2d). The increase in the perimeter was also higher in LPS-treated PTN-Tg mice (Fig. 2e). The decrease caused by LPS in the circularity index was more pronounced in PTN-Tg mice (Fig. 2f). Overall, the data demonstrate that LPS-induced microglial response is increased in the PFC of PTN-Tg mice compared to that of WT mice. Interestingly, we observed that the morphological changes induced by LPS in microglial cells of both genotypes were not prevented by the previous administration of the TLR4 antagonist TAK-242 (Fig. 2, TAK-LPS).

**Fig. 3:**
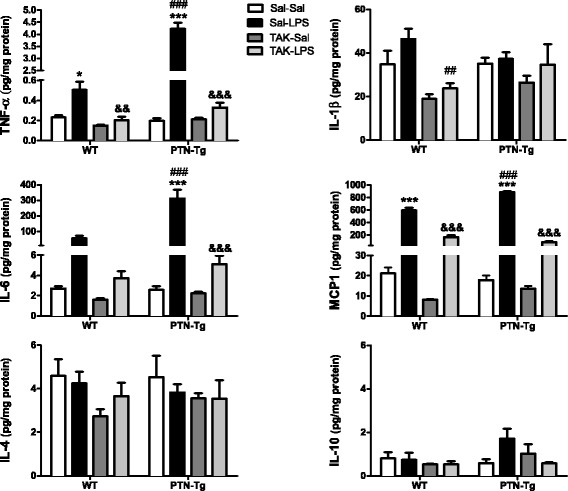
Effects of TAK-242 and LPS on cytokine expression in the PFC of PTN-Tg mice. TNF-α, Il-1β, IL-6, MCP-1, IL-4, and IL-10 protein levels measured using a Milliplex system in PFC of mice pretreated with saline (Sal) or TAK-242 (TAK) and treated with saline (Sal) or LPS (*n* = 5/group). *P < 0.05, ***P < 0.001 vs. Sal + Sal within the same genotype. ^##^ *P* < 0.01, ^###^ *P* < 0.001 vs. WT within the same treatment. &&*P* < 0.01, &&&*P* < 0.001 vs. Sal + LPS within the same genotype. *SAL* saline, *LPS* lipopolysaccharide, *TAK* TAK-242, *WT* wild-type mice, *PTN-Tg* mice with transgenic pleiotrophin overexpression in the brain, *TNF* Tumor necrosis factor-α, *IL-1β* interleukin 1β, *IL-6* interleukin 6, *MCP-1* monocyte chemoattractant protein-1, *IL-4* interleukin 4, *IL-10* interleukin 10

We measured the contents of different cytokines in the PFC of mice from both genotypes. Confirming the neuroimmune response to LPS administration, we found that the levels of proinflammatory cytokines such as TNF-α, IL-6, and MCP-1 were many-fold upregulated in the PFC of LPS-treated WT mice compared to those of the saline-treated group (Fig. 3, Sal-LPS vs. Sal-Sal). More importantly, we found a highly significant ~7-fold upregulation of the levels of TNF-α in the PFC of LPS-treated PTN-Tg mice compared to that of WT mice (Fig. 3). Similarly, the levels of IL-6 in the PFC of LPS-treated PTN-Tg mice were increased compared to those of WT mice (Fig. 3). Interestingly, pretreatment with TAK-242 efficiently blocked LPS-induced increases of TNF-α and IL-6 in the PFC of mice from both genotypes (Fig. 3, Sal-LPS vs. TAK-LPS). In addition, we found significantly higher levels of MCP-1 in the PFC of LPS-treated PTN-Tg mice compared to those of WT mice although, in this case, LPS effect on MCP-1 levels was partially blocked by previous administration of TAK-242 in both genotypes (Fig. 3). In contrast, we did not observe relevant genotypic differences in the levels of IL-1β (Fig. 3). Concerning the anti-inflammatory cytokines IL-10 and IL-4, we did not find genotype- or treatment-related significant differences in their contents in the PFC of LPS-treated PTN-Tg mice compared to those of WT mice (Fig. 3).

Overall, the data demonstrate that LPS-induced microglial activation is enhanced in the PFC of PTN-Tg mice compared to that of WT animals. Interestingly, the data suggest that TLR4 activation is crucial for LPS-induced cytokine release in both genotypes but does not play a significant role in LPS-induced microglial morphological response in either genotype.

### Effects of TAK-242 and LPS in the striatum of WT and PTN-Tg mice

**Fig. 4:**
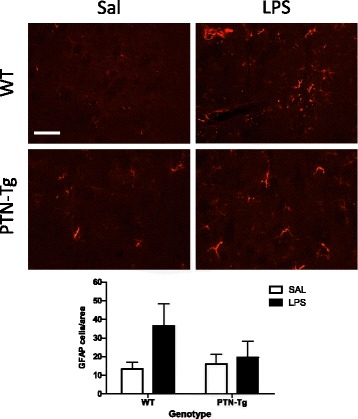
LPS effects on astrocytosis in the striatum of WT and PTN-Tg mice. Photomicrographs are from GFAP-immunostained striatal sections of saline + saline (Sal)- or saline + LPS (LPS)-treated animals (*n* = 4–5/group). The graph represents quantification of data obtained from the counts of GFAP-positive cells in the striatum. Scale bar = 200 μm. *SAL* saline, *LPS* lipopolysaccharide, *WT* wild-type mice, *PTN-Tg* mice with transgenic pleiotrophin overexpression in the brain, *GFAP* glial fibrillary acidic protein

**Fig. 5:**
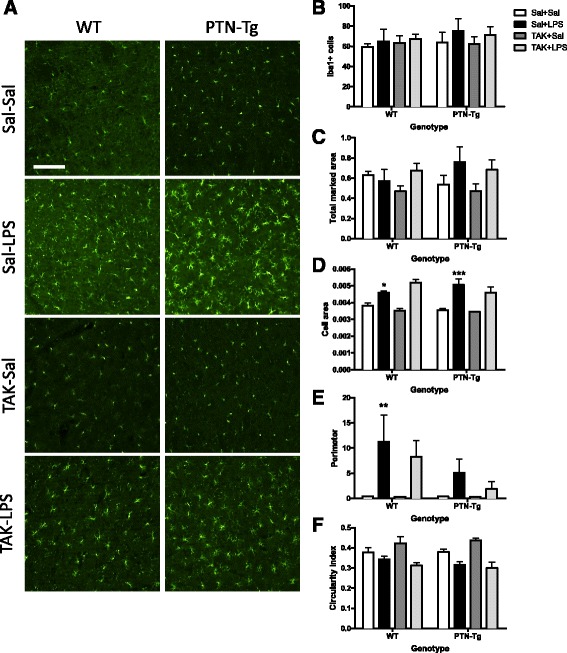
Effects of TAK-242 and LPS on microglia activation in the striatum of PTN-Tg mice. Photomicrographs are from Iba-1-immunostained striatal sections of saline (Sal)- or TAK-242 (TAK)-pretreated and saline (Sal)- or LPS-treated animals (*n* = 4–5/group) (**a**). Graphs represent quantification of data (mean ± SEM) obtained from the counts of Iba-1-positive cells (**b**), total marked area (**c**), cell area (**d**), soma perimeter (**e**), and circularity index (**f**) in the striatum. *P < 0.05, **P < 0.01, ***P < 0.001 vs. Sal + Sal within the same genotype. Scale bar = 100 μm. *SAL* saline, *LPS* lipopolysaccharide, *TAK* TAK-242, *WT* wild-type mice, *PTN-Tg* mice with transgenic pleiotrophin overexpression in the brain, *Iba1* ionized calcium-binding adaptor molecule 1

To investigate the effects of a more modest PTN overexpression (~20%, PTN-Tg mouse striatum) [28] on LPS-induced neuroinflammation, we tested the astrocytic and microglial response in striatal sections of WT and PTN-Tg mice treated with saline (Sal-Sal) or LPS (Sal-LPS). We did not find genotypic- or treatment-related significant differences in the number of GFAP+ cells between groups (Fig. 4). Similarly to PFC, LPS treatment tended to increase the number of GFAP+ cells in the striatum of WT mice, whereas this tendency was absent in PTN-Tg mice (Fig. 4). Concerning microglia (Fig. 5), immunohistochemistry for Iba1 did not reflect significant changes in the number of Iba1+ cells or in the total marked area in the striatum of WT and PTN-Tg mice after LPS treatment (Fig. 5a, b, c). However, signs of hypertrophism, soma enlargement, and sprouting of new ramifications in LPS-treated animals were observed in both genotypes compared to those in saline-treated groups (Fig. 5a). The Iba1+ cell area was significantly increased in both genotypes but especially in LPS-treated PTN-Tg mice compared to that in WT mice (Fig. 5d). On the other hand, the increase in the perimeter was higher in LPS-treated WT mice (Fig. 5e), whereas no differences between groups were found in the case of the circularity index (Fig. 5f). We did not observe significant effects of pretreatment with TAK-242 on LPS-induced effects on microglia (Fig. 5). Overall, the data demonstrate that LPS-induced microglial response in the striatum is more modest than that in the PFC and that the moderate PTN overexpression in this brain area of PTN-Tg mice does not play a significant modulatory role on LPS effects.

### Pleiotrophin potentiates NO production in LPS-stimulated BV2 cells

**Fig. 6:**
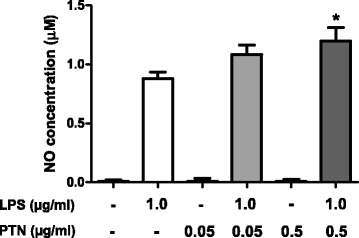
Effects of PTN on LPS-induced NO production in BV2 microglial cells. Cells were treated with the indicated concentrations of PTN (0.05 or 0.5 μg/ml) and/or with LPS (1.0 μg/ml) for 24 h. The data of the levels of NO in the media are expressed as the mean ± SEM. *P < 0.05 vs. cells treated with LPS. *NO* nitric oxide, *LPS* lipopolysaccharide, *PTN* pleiotrophin

To test the possible direct effects of PTN on murine microglia in vitro, we investigated its effects on the production of NO, which is a key inflammatory mediator in LPS-stimulated BV2 microglia. First, we did not detect any effect in BV2 cells incubated with PTN alone (0.05 and 0.5 μg/ml, Fig. 6). LPS (1.0 μg/ml) induced a significant increase in the production of NO compared with control cells (Fig. 6). Concomitant incubation of BV2 cells with LPS (1.0 μg/ml) and PTN (0.5 μg/ml) significantly potentiated the production of NO compared to cells only treated with LPS (Fig. 6).

## Discussion

Pleiotrophin is a cytokine that is found highly upregulated in diverse pathologies of the CNS characterized by overt neuroinflammation including neurodegenerative diseases, addictive disorders, ischemia, and neuropathic pain [6, 38, 39]. The goal of this study was to investigate whether pleiotrophin regulates the astrocytic response and the activation of microglia in the brain after an inflammatory challenge. We used the systemic LPS treatment acute inflammation murine brain model. In the in vivo study, we found that LPS induced a moderate increase in the number of GFAP+ astrocytes in the PFC and striatum of WT mice. After LPS treatment, astrocytes appear hypertrophic, particularly in the PFC, suggesting a LPS-induced upregulation of GFAP protein concentrations in astrocytes of WT mice. This response was significantly reduced in the PFC of LPS-treated PTN-Tg mice, an area with a more significant PTN overexpression in this genotype [24]. We previously found a small (~20%) increase of striatal GFAP+ astrocytes in amphetamine-treated PTN knockout (PTN-/-) mice which could be attributed to compensatory mechanisms [19, 21]. Interestingly, we recently found a highly significant (13-fold) increase in the number of GFAP+ astrocytes in amphetamine-treated PTN-Tg mice suggesting an enhanced neuroinflammatory response induced by amphetamine in the presence of higher levels of PTN [19]. Taking together, these data suggest that PTN differentially regulates the astrocytic response depending on the inflammatory stimulus.

Although upregulation of GFAP in astrocytes is considered an indicator of reactive astrogliosis and neuroinflammation, the regulation by PTN of two key elements of neuroinflammation, microglia activation, and cytokines release had not been tested before. In WT mice, LPS tended to increase the perimeter of Iba1+ cells and induced a significant increase in these cells area in the PFC, indicating the presence of hypertrophic microglia. In addition, LPS induced significant increases in the levels of TNF-α, IL-6, and MCP-1 in WT mice confirming LPS-induced neuroinflammation in normal mice. Interestingly, we found that PTN overexpression enhances microglial cell morphological changes to an activated-form in the PFC of LPS-treated PTN-Tg mice compared to that of WT mice. This was accompanied by more pronounced LPS-induced increases of TNF-α, IL-6, and MCP-1 in PTN-Tg mice compared to WT animals, indicating an important role of PTN on microglial activation and neuroinflammation. Interestingly, the TLR4 antagonist TAK-242 did not have any effect on LPS-induced microglia morphological changes in either genotype but efficiently blocked LPS-induced increases of TNF-α, IL-6, and MCP-1 in both genotypes. This is the first study showing that PTN overexpression in vivo significantly potentiates LPS-induced microglial activation and neuroinflammation. It is important to note that we did not find differences between genotypes in saline-treated animals suggesting that PTN is an important cytokine in the promotion of the effects triggered by the inflammatory stimulus but does not trigger proinflammatory cascades itself. Accordingly, our studies in vitro in murine BV2 microglia cells demonstrate that incubation with PTN alone does not cause significant effects on microglia. However, PTN significantly enhanced LPS-induced NO release in BV2 cells. Taking together, these studies may be of relevance since PTN is found highly upregulated in different brain disorders characterized by neuroinflammation such as Parkinson’s disease [16], Alzheimer’s disease [40], addictive disorders [14, 41, 42], tumors [43], and ischemia [44]. However, we must be cautious because the mechanisms involved in the short-term inflammatory response induced by LPS might differ substantially from those involved in the long-term inflammation associated with the brain disorders mentioned above. In the present work, we have demonstrated for the first time that PTN potentiates microglial activation and neuroinflammation after an acute exposure to the inflammatory stimulus. Additional studies are needed to describe its role in chronic conditions of neuroinflammation.

Inflammation is a host defense mechanism mounted to reduce injury. However, prolonged or uncontrolled inflammation can lead to tissue damage and destruction. On the other hand, recent studies have shown that activated microglia contributes to the maintenance of tissue homeostasis and protection of the CNS under various pathological conditions [45]. Whether microglial activation primed by elevated levels of PTN contributes to the deleterious or homeostatic effects of microglia needs to be clarified. However, it has to be noted that one of the mechanisms that microglia adopts to downregulate inflammation is the production of anti-inflammatory mediators such as IL-4 and IL-10, which suppress the function of proinflammatory cytokines [46]. Here, we demonstrate that the content of IL-10 and IL-4 is not affected by increased PTN levels in the PFC of PTN-Tg mice, whereas the proinflammatory cytokines TNF-α, IL-6, and MCP-1 are highly upregulated compared to WT mice treated with LPS. Thus, our data suggest that high PTN levels lead activated microglia to promotion of neuroinflammation rather than suppression.

The mechanism of action of PTN supports our findings. PTN binds receptor protein tyrosine phosphatase (RPTP) β/ζ (a.k.a. PTPRZ1) [47] and inactivates its phosphatase activity. Inhibition of the phosphatase activity of RPTPβ/ζ by PTN binding regulates the tyrosine phosphorylation of substrates of RPTPβ/ζ that are known regulators of neuroinflammation such as Fyn kinase [48]. After stimulation with LPS, Fyn is activated in microglia by increased phosphorylation in Y416 [48]. Activated Fyn phosphorylates PKCδ at Y311, contributing to an increase in its kinase activity. The Fyn-PKCδ-signaling axis further activates the LPS-induced MAP kinase phosphorylation and activation of the NFκB pathway, implying that Fyn is a major upstream regulator of proinflammatory signaling [48]. These signaling events are observed as well in animal models of PD [48]. Previously, we demonstrated that PTN is a key modulator of tyrosine phosphorylation of Fyn [49], suggesting that PTN could be a major upstream regulator of microglial neuroinflammatory processes in all those neurological disorders in which significantly increased levels of PTN have been detected by triggering inflammogen-induced increase in Fyn kinase activity.

## Conclusions

Our findings identify for the first time that PTN is a novel and potent regulator of neuroinflammation. Pleiotrophin potentiates LPS-stimulated microglia activation. Our results indicate that the regulation of PTN signaling pathways may constitute new therapeutic opportunities particularly in those neurological disorders characterized by increased PTN cerebral levels and neuroinflammation.

## Abbreviations

ANOVA: Analysis of variance
BBB: Blood-brain barrier
BCA: Bicinchoninic acid
CNS: Central nervous system
COX2: Cyclooxygenase-2 enzyme
GFAP: Glial fibrillary acidic protein
Iba1: Ionized calcium-binding adaptor molecule 1
IL-10: Interleukin 10
IL-1β: Interleukin 1β
IL-4: Interleukin 4
IL-6: Interleukin 6
iNOS: Inducible nitric oxide synthase
LPS: Lipopolysaccharide
MAP kinase: Mitogen-activated protein kinase
MCP-1: Monocyte chemoattractant protein-1
NFκB: Nuclear factor-κB
NO: Nitric oxide
PD: Parkinson’s disease
PFC: Prefrontal cortex
PKCδ: Protein kinase C delta type
PTN-/-: PTN genetically deficient mice
PTN: Pleiotrophin
PTN-Tg: Mice with transgenic PTN overexpression in the brain
qRT-PCR: Quantitative real-time polymerase chain reaction
RPMI-1640: Roswell Park Memorial Institute 1640 medium
RPTP β/ζ (PTPRZ1): Receptor protein tyrosine phosphatase β/ζ
SEM: Standard error of the mean
TLR4: Toll-like receptor 4
TLRs: Toll-like receptors
TNF-α: Tumor necrosis factor-α
WT: Wild type
